# Rasch Validation of the Arabic Version of the Behavioral Intention to Interact With Peers With Intellectual Disability Scale

**DOI:** 10.3389/fpsyg.2019.02345

**Published:** 2019-10-16

**Authors:** Ghaleb H. Alnahdi

**Affiliations:** Department of Special Education, College of Education, Prince Sattam Bin Abdulaziz University, Al-Kharj, Saudi Arabia

**Keywords:** disability, rasch analysis, children, arabic, intention to interact, intellectual disability

## Abstract

This study aimed to examine the construct validity of the Arabic version of the behavioral intention to interact with peers using an intellectual disability (ID) scale. Rasch analysis was used to examine the psychometric properties of the scale. The sample contained 290 elementary students in Saudi Arabia (56% were girls and 44% were boys). Several parameters were examined: overall fit, item fit, person fit, assumption of local independence, and the scale’s unidimensionality. Eight items were rescored, 22 misfit persons were removed, and no item with differential item functioning (DIF) was detected. Disordered thresholds were detected in eight items. The scale demonstrated good internal consistency [person separation index (PSI) 0.80] and fulfilled all the requirements of the Rasch model. After rescoring the eight items, Rasch analysis supported the scale’s unidimensionality to measure children’s behavioral intention to interact with peers with ID. The Arabic version of the scale, with the proposed scoring, could be a useful tool to measure children’s behavioral intention to interact with peers with ID. Further studies with different samples are warranted to confirm the study’s findings.

## Introduction

Inclusion of students with disabilities in schools is a main change that has occurred in the handling of childhood disabilities in recent years (Rosenbaum, 2010). A positive attitude toward individuals with disabilities is one of the most important factors ensuring the success of this transformation. Behavioral intention is one of three components of attitude along with the affective and cognitive components (Eagly and Chaiken, 1998).

Accurately measuring children’s intention and willingness requires a scale tested on different samples and with proven psychometric properties. This challenge is greater with students in Arab societies, including Saudi Arabia. There is a significant lack of measurement tools for special education (Suleiman et al., 2011) with approved validity and reliability coefficients in Arab environments. Applying item response theory to validate the structure of scales and to examine their psychometric properties will help to provide Arab measurement resources that can be used to examine the effectiveness of interventions designed to promote students’ intention to interact with peers with intellectual disability (ID). Few measures have attempted to study behavioral intention separate from the cognitive and emotional domains. For example, the Chedoke-McMaster Attitudes toward Children With Handicaps (CATCH) scale, one of the scales most commonly used to measure children’s attitudes, measures behavioral intention as one of three subscales. Another scale was developed to measure children’s behavioral intention to interact with peers with ID in 2007 (Siperstein et al., 2007). The behavioral intention to interact with peers with ID (BIS) (Siperstein et al., 2007) was developed with two subscales: intention to interact in school and intention to interact outside of school. Children with high scores on this scale are interpreted as willing to interact with peers with ID.

The English version of the BIS has good psychometric properties; Cronbach’s α was 0.932 for the scale overall, 0.891 for the outside-school subscale, and 0.872 (Siperstein et al., 2007) and 0.930 (Brown et al., 2011) for the in-school subscale. For the Arabic version, Cronbach’s α was 0.928 for the scale overall, 0.861 for the outside-school subscale, and 0.905 for the in-school subscale (Alnahdi and Schwab, unpublished). Although the BIS was tested with various samples from different countries, including the United States (Siperstein et al., 2007), Canada (Brown et al., 2011), Saudi Arabia (Alnahdi and Schwab, unpublished), and Greece (Giagazoglou and Papadaniil, 2018), its psychometric properties were examined based on the classical test theory approach only. Rasch analysis provides a powerful tool for examining the specific psychometric properties of a scale (Smith, 2001; Smith et al., 2002; Tennant and Conaghan, 2007; Lee et al., 2010). Fitting the observed data to the Rasch model offers an alternative method for examining the scale’s construct validity. For instance, it provides distinct parameters and statistics for each item and orders the items based on how difficult it is to be endorsed by participants. In addition, it allows examination of whether any items are biased according to any subgroup such as gender. Furthermore, it allows examination of the scales’ scoring structure and whether it is working as expected (Alnahdi, 2019). It also enables transformation of the ordinal data into interval data (Tennant and Conaghan, 2007). Thus, this study aims to examine the construct validity of the Arabic version of the BIS scale using Rasch analysis.

## Materials and Methods

### Rasch Analysis

This research followed Tennant and Conaghan’s (2007) guidelines on using and reporting the results of Rasch analysis. Analyses were conducted using the RUMM2030 software (Andrich et al., 2010). There are two types of Rasch models that can be used. The partial credit model (default model in RUMM2030) used in this study is based on the significant likelihood ratio test, for which the rating scale is recommended as with the non-significant likelihood ratio test (Tennant et al., 2011; Vincent et al., 2015). In the partial credit model, the thresholds are estimated for each item (Andrich and Marais, 2019).

Overall statistics were first checked by looking for non-significant item-trait interaction chi-square, as in case of significant chi-square that would indicate that “the hierarchical ordering of the items varies across the trait, compromising the required property of invariance” (Tennant and Conaghan, 2007, p. 1360). In addition, item residual mean close to zero, and a standard deviation close to 1 (Alnahdi, 2018), would be an indicator for normally distributed residuals. Threshold plots were checked for items with disordered thresholds, to be rescored by combining adjacent categories.

**FIGURE 1:**
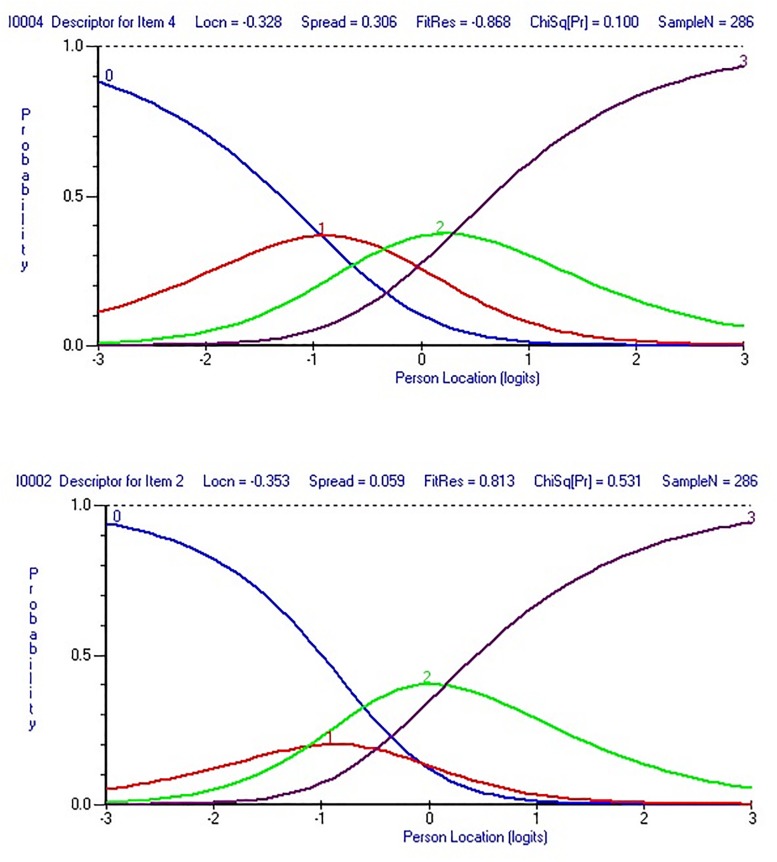
Category probability curves for item 2 with threshold disorder **(bottom)** and item 4 with no threshold disorder **(top)**.

**FIGURE 2:**
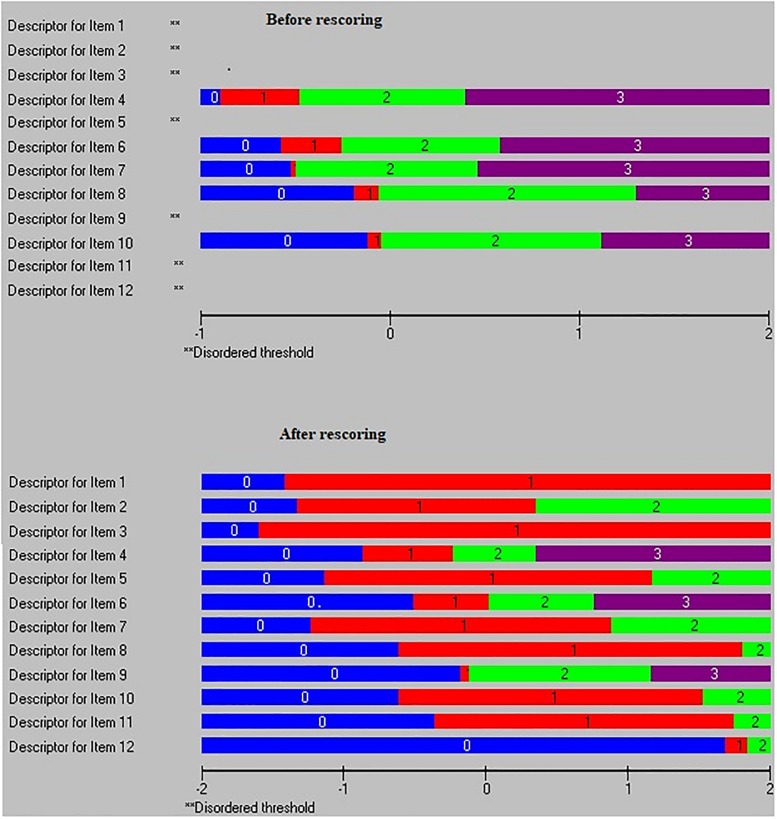
Threshold map before and after rescoring eight items with threshold disorder.

This step was conducted through the category characteristic curve for each item and the threshold map for all items at once. Items that showed disordered thresholds were rescored to combine adjacent categories in order to correct the disorder (Tennant et al., 2004; Tennant and Conaghan, 2007). “For a well-fitting item you would expect that, across the whole range of the trait being measured, each response option would systematically take turns showing the highest probability of endorsement” (Pallant and Tennant, 2007, p. 6). In other words, “items with a response option that never takes its turn having the highest probability at any point would be an indicator of threshold disorder” (Alnahdi, 2019, p. 105). This property can be checked visually using the category characteristic curve (see Figure 1 for an example of an item before and after rescoring and how the threshold disorder was clear before the rescoring). In addition, the RUMM2030 software provides an easy-to-read map by highlighting and removing all items with a threshold disorder from the threshold map (see Figure 2 for how the threshold map contains all the items after rescoring items with the disorder threshold).

Persons exceeding the ±2.5 person-fit residual range were removed (Tennant and Conaghan, 2007), and item-fit residual statistics were checked to identify items exceeding the acceptable ±2.5 range (Tennant and Conaghan, 2007). Correlations between item residuals were checked to identify issues related to local dependency. Item residuals in a unidimensional scale would not show high correlation because the Rasch factor that clustered them was removed. A value of 0.30 above the average of the residual correlations of all items is considered high, and could indicate violation of the local dependency assumption (Christensen et al., 2017).

The unidimensionality of the scale was examined via Smith’s test of unidimensionality, implemented in RUMM2030. Two ability estimates for each individual were computed after running a principal component analysis (PCA) of the residuals; an independent *t*-test then examined whether the two ability estimates (one from items with positive loadings on the first PCA component, and the other from items with negative loadings) had significant differences. Significant tests should not exceed 5% of the sample or the lower limit of the binomial 95% confidence interval of proportions at the 5% level or less (Smith, 2002; Tennant and Conaghan, 2007; Hadzibajramovic et al., 2015; Alnahdi, 2018). This means that there are only significant differences between the two estimates of participants in 5% or less of the cases, and 95% or more of the cases show no differences between the two estimates. This is an indicator of there being one dimension that clusters the data together.

Differential item functioning (DIF) was checked to ensure that items functioned the same way regardless of participants’ gender or age (Pallant and Tennant, 2007), and a value of 0.7 or higher on the person separation index (PSI) indicated good internal consistency of the scale (Tennant and Conaghan, 2007). The final step was to transform the raw scores to interval scores, which are easier to interpret because any change in one unit has the same weight across the scale (Alnahdi, 2018). This not true for raw scores, change in one unit of which would have different weights across the scale.

### Sample and Instrument

The sample consisted of 290 elementary students from 4th to 6th grade, of whom 162 (56%) were girls and 128 (44%) were boys. This study was approved by the institutional review board (IRB) of Prince Sattam bin Abdulaziz University. After written and informed consent was obtained from the children’s parents, a pencil and paper questionnaire was distributed to students in the classroom after school by their teachers. The Arabic version of the BIS scale was previously translated (Alnahdi and Schwab, unpublished) and showed good reliability (0.928), and good fit indices in the confirmatory factor analysis (CFA) are a good indicator that the observed data fit the hypothesized two-factor model from the English version of the scale. The scale contains 12 items, with six items on the intention to interact in school subscale and six items on the intention to interact outside of school subscale. Four Likert options were provided for each item, from strongly disagree and disagree to agree and strongly agree (Siperstein et al., 2007).

## Results

In the first analysis with the sample of 290 participants, chi-square for item-trait interaction was significant [χ^2^118.93(48) = *p* < 0.05], which did not support the overall fit. Additionally, 8.8% of *t*-tests were significant when testing for unidimensionality, above the recommended limit of 5% (see Table 1). The lower limit of the 95% CI for the binominal test was also higher than 5% (7.3%). The threshold plot was checked, and eight items were found to have threshold disorder.

**TABLE 1:** Rasch statistics for each run.

		**Item residual fit**		**Person residual fit**		**Item-trait interaction**			**Unidimensionality *t*-tests**	
					
	***N***	**Mean**	***SD***	**Mean**	***SD***	**χ^2^(*df*)**	***p***	**PSI**	**% significant tests**	**Lower limit of 95% CI**
Initial analysis	290	0.17	2.228	–0.30	1.33	118.93 (48)	0.000	0.808	8.79%	7.31%
After rescoring eight items	290	–0.05	1.609	–0.39	1.26	65.82 (48)	0.044	0.812	6.67%	4.81%
After removing 22 misfit persons	268	–0.07	1.516	–0.29	1.011	58.18 (48)	0.149	0.805	6.82%	4.91%
Ideal values		0.0	<1.4	0.0	<1.4		>0.05	>0.7	≤5%	≤5%

*CI = confidence interval in the binomial test of proportions; PSI = person separation index; SD = standard deviation.*

In the second analysis, eight items were rescored by combining adjacent categories with disorder. Figure 1 shows an example of item-level improvement after rescoring items with threshold disorder. Figure 2 shows improvement of the threshold map after rescoring the eight items.

In the third analysis, 22 persons with a fit residual outside of the ±2.5 range were removed. In this run, chi-square for item-trait interaction was non-significant for the first time [χ^2^58.18(48) = *p* = 0.149], a positive indicator for overall goodness of fit.

**FIGURE 3:**
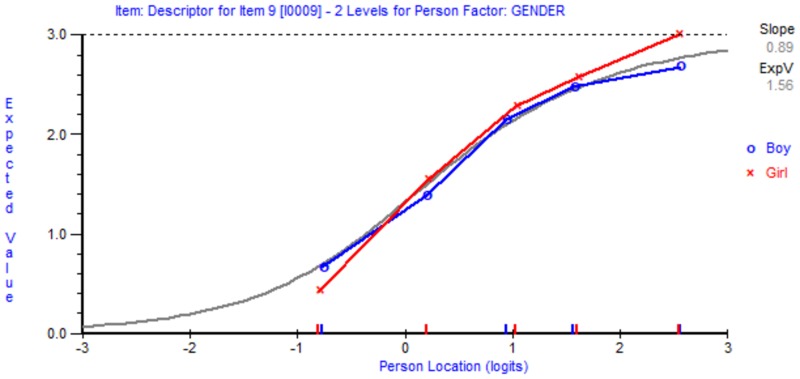
Item characteristic curves showing item 9 as an example of an item with no differential item functioning (DIF).

Next, DIF analysis was conducted to examine whether all items functioned similarly regardless of participants’ age or gender. The findings showed no indictors of DIF in any of the items. Figure 3 shows an example of item 9 with no DIF effect by gender. As the figure shows, participants from both genders with similar levels of intention to interact with peers with ID (person location/*x*-axis) responded similarly to item 9 in comparison with the Rasch model expectation (gray line in the figure).

Table 2 shows the scale with the new scoring applied. Two items that were scored as 0011, five as 0112, one as 0012, and the remaining four as 0123. Table 3 shows the statistics for each item in the scale. Items were sorted based on location, from most to least difficult to endorse with the lowest location value.

**TABLE 2:** BIS items with new scores.

**Item**		**Strongly disagree**	**Disagree**	**Agree**	**Strongly agree**
1	**Lend a student with ID a pencil or pen**	0	0	1	1
2	**Stand next to a student with ID while waiting in line**	0	1	1	2
3	**Go up to a student with ID and say hello**	0	0	1	1
4	Talk to a student with ID during free time or lunch	0	1	2	3
5	**Choose a student with ID to be on your team in gym class**	0	1	1	2
6	Work with a student with ID on a project in class	0	1	2	3
7	**Sit next to a student with ID on the bus for a field trip**	0	1	1	2
8	**Spend time with a student with ID outside of school**	0	1	1	2
9	Invite a student with ID to go out with you and your friends	0	1	2	3
10	Invite a student with ID to your home	0	1	2	3
11	**Go to the movies with a student with ID**	0	1	1	2
12	**Talk about personal things with a student with ID**	0	0	1	2

*ID = intellectual disabilities. Bold text indicates rescored items.*

**TABLE 3:** Item fit statistics.

**Item**	**Location**	***SE***	**Fit residual**	**χ^2^**	***p*^a^**
12	1.713	0.096	1.53	10.509	0.033
11	0.678	0.105	1.042	0.839	0.933
8	0.609	0.107	–2.346	15.148	0.004
10	0.467	0.079	0.749	1.365	0.850
9	0.271	0.081	–1.915	6.211	0.184
6	0.12	0.082	–0.397	2.574	0.631
5	0.109	0.109	–1.441	7.948	0.094
7	–0.15	0.11	–1.454	6.909	0.141
4	–0.266	0.086	0.226	5.701	0.223
2	–0.551	0.115	2.147	4.914	0.296
1	–1.365	0.193	1.621	8.865	0.065
3	–1.635	0.208	–0.007	1.953	0.744

*SE = standard error. ^*a*^Bonferroni adjusted *p* = 0.00416 (0.05/12).*

A challenge in interpreting scale scores is to understand the differences between them and to know whether progress or change of one unit in score is weighted equally across the scale. For example, is the change in score from 10 to 11 equivalent to the change from 16 to 17? When using raw or logit scores, the change is not equivalent. Thus, raw scores from the Rasch analysis were converted to interval scores, in which a difference of one unit has the same weight across all scores, using the following formula: “Y = M + (S × logit score). S = range of interval-level scale [(60; for a 0 to 60 scale)] divided by the actual range of logit scores, and M = (minimum score of interval-level scale) – (minimum logit score × S)” (Alnahdi, 2018, 355). Table 4 shows the transformation from each raw score to the equivalent value in interval score.

**TABLE 4:** Transformation table for conversion of BIS total raw ordinal-level score to interval-level score.

**Raw score**	**Interval-level score**	**Raw score**	**Interval-level score**
0	0.0	14	46.8
1	10.2	15	48.1
2	17.5	16	49.3
3	22.7	17	50.4
4	26.8	18	51.6
5	30.1	19	52.7
6	32.9	20	53.7
7	35.3	21	54.8
8	37.4	22	55.9
9	39.3	23	56.9
10	41.0	24	57.9
11	42.6	25	59.0
12	44.1	26	60.0
13	45.5		

**FIGURE 4:**
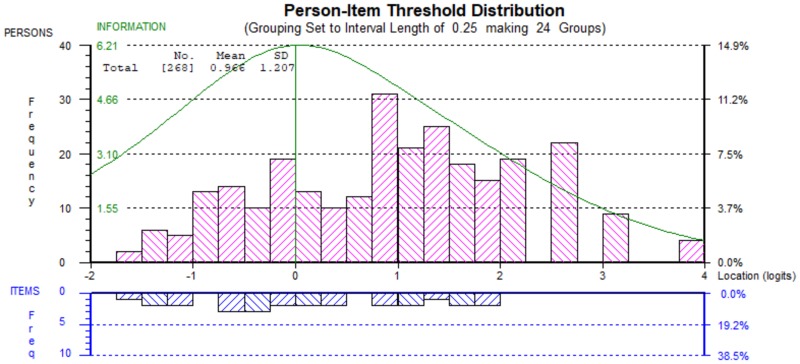
Person-item threshold plot of the modified 12-item BIS showing distribution of students’ intention to interact with peers with intellectual disability estimates **(top)** and item thresholds **(bottom)**. The curve represents the information function of the scale.

The person-item threshold plot (Figure 4) showed a positive mean location for the sample (0.966), indicating that the sample as a whole had a higher level of intention to interact with peers with ID compared to the average of item difficulties (Tennant and Conaghan, 2007). As the figure shows, most frequencies of persons’ locations were to the right side of the plot (from point zero). Additionally, aside from those with very high ability, the plot showed an acceptable spread of items thresholds covering the spread of students’ intention to interact with peers with ID, which can indicate good targeting of the Arabic BIS (Alnahdi, 2018). The Arabic BIS showed good internal consistency, with a value greater than 0.7 (0.88) (Tennant and Conaghan, 2007). In sum, the modified Arabic BIS has good psychometric properties, fitting the unidimensional Rasch model, for measuring children’s intention to interact with peers with ID. Therefore, these new scores could be used to score participants’ responses to the Arabic BIS. Options for children should be the same as the four Likert options. The change would be in how to score these four options. For example, if a student chooses strongly disagree or disagree on item 1 will score (0), and if he/she choose agree or strongly agree will score as (1). While this will be different for item 12 where student will get 2 if strongly agree was chosen. In another word, agreeing with item 12 has more weight on the total score than item1. The total scores for students should be reported in interval level scores as shown in Table 4.

## Discussion

This study aimed to examine the construct validity of the Arabic version of the BIS using Rasch analysis. The first run of the Rasch model indicated that the Arabic version of the BIS was not unidimensional. Eight items showed categorical issues with threshold disorder; the items were rescored to resolve this issue. Rescoring resulted in a better uniform spread of the thresholds plot.

Item 12, “Talk about personal things with a student with ID,” was the most difficult item to endorse in the scale. This means that students agreeing with this item have a higher positive intention to interact with peers with ID. This finding is consistent with the research of Siperstein et al. (2007) and with Brown et al. (2011), in which this item has the lowest mean score overall.

Item 3 (“Go up to a student with ID and say hello”), item 1 (“Lend a student with ID a pencil or pen”), and item 2 (“Stand next to a student with ID while waiting in line”) were the easiest items to endorse. Similarly, these three items were the highest endorsed in Siperstein et al. (2007) and Brown et al. (2011).

Response dependency is one the reasons that might affect the unidimensionality of scales (Tennant and Conaghan, 2007). Residual correlations were examined to identify response dependency between items after removing the Rasch factor (behavioral intention). No issues were detected, based on the finding of residual correlations 0.30 higher than the average of all items (Tennant and Conaghan, 2007). Because this was a positive indicator of the local independence of items, no further action was needed (Tennant and Conaghan, 2007).

After rescoring eight items in the second run to correct item threshold disorder, and after removing 22 misfit persons with residuals out of the ±2.5 range, the lower limit of the 95% CI for the binominal test was less than 5% (4.9%), indicating unidimensionality.

The items of the Arabic BIS were invariant (no DIF effect), suggesting that students with a similar level of intention to interact with peers with ID would score the same, regardless of age and/or gender (Tennant and Conaghan, 2007; Alnahdi, 2018). The transformation table from raw score to interval score is important in understanding the differences between the two scores retrieved from the Arabic BIS. For example, improvement by one unit in raw score from 10 to 11 is equivalent to a mean improvement of 1.6 units in interval score, from 41 to 42.6. In contrast, change by one unit in raw score from 16 to 17 is equivalent to an improvement of 1.1 units in interval score, from 49.3 to 50.4. This indicates the importance of using interval-level scores, in which a change of one unit has equal weight across all scores.

This study provides a measurement tool for researchers in the Arab region with interest in children’s attitudes, intention, and behaviors toward individuals with ID. This is especially important in light of the shortage of validated measures that have been tested on different samples. The importance of this study is further enhanced by the fact that few scales have been developed in Arabic and tested on different samples, especially those that focus on issues related to individuals with disabilities. Examining this scale with different samples in the Arab region, and in other regions of Saudi Arabia, will provide a better understanding of the scale’s psychometric properties.

## Data Availability Statement

The datasets analyzed in this manuscript are not publicly available. Requests to access the datasets should be directed to GA, ghalanhdi@gmail.com.

## Author Contributions

The author confirms being the sole contributor of this work and has approved it for publication.

## Conflict of Interest

The author declares that the research was conducted in the absence of any commercial or financial relationships that could be construed as a potential conflict of interest.
